# Significance of resistin expression in acute pancreatitis

**DOI:** 10.3892/etm.2015.2270

**Published:** 2015-02-06

**Authors:** LE-NING XUE, XIAO-YONG WANG, YONG TAN, MIN LIN, WEI ZHANG, KE-QUN XU

**Affiliations:** Department of Gastroenterology, Changzhou Second Hospital Affiliated to Nanjing Medical University, Changzhou, Jiangsu 213003, P.R. China

**Keywords:** acute pancreatitis, resistin, C-reactive protein, tumor necrosis factor-α, interleukin-1β

## Abstract

The aim of the present study was to detect the expression of resistin in rats with acute pancreatitis (AP) and investigate its significance in the pathogenesis of AP. In total, 40 Sprague-Dawley rats were randomly divided into four groups (n=10), including the normal control, sham-operated, acute edematous pancreatitis (AEP) and acute necrotizing pancreatitis (ANP) groups. Following the establishment of animal models, the levels of serum resistin, C-reactive protein (CRP), tumor necrosis factor-α (TNF-α) and interleukin (IL)-1β were measured using ELISA. Resistin expression in the pancreatic tissues was detected using an immunohistochemical method. In addition, the mRNA expression of resistin in the pancreatic tissues was analyzed with quantitative polymerase chain reaction. The levels of serum amylase, serum resistin, TNF-α, IL-1β and CRP were all significantly higher in the AEP and ANP groups when compared with the control and sham-operated groups (P<0.01), as were the pancreas/body weight ratios and pathological scores of the pancreas. These increases were more significant in the ANP group than in the AEP group (P<0.05). The mRNA expression levels of resistin in the pancreatic tissues were markedly higher in the AEP and ANP groups when compared with the control and sham-operated groups (P<0.01), particularly in the pancreatic tissues of the ANP group, which exhibited notably higher levels compared with the AEP group. The serum resistin level was found to positively correlate with the serum levels of CRP, TNF-α and IL-1β, and the pathological scores of the pancreatic tissues. In conclusion, the results indicated that resistin may be associated with the occurrence and development of AP; thus, the protein may be a valuable indicator for assessing the severity of AP.

## Introduction

The incidence of pancreatitis is markedly increased with the improvement of living conditions, particularly acute pancreatitis (AP). AP is a common clinical disease, and one of the main causes of it is hypertriglyceridemia (1). The prognosis of the majority of cases of AP is good; however, some types, particularly severe AP, may lead to multiple organ failure, resulting in serious adverse consequences for the patient (2). Currently, the cause and mechanisms of AP are not completely clear, and there is no specific drug used to treat AP. The predominant treatment method is comprehensive therapy (3). It is believed that AP may be closely associated with obesity, trypsin activation, inflammatory mediator activation, pancreatic blood circulation disorder, pancreatic cell apoptosis and other factors (4,5). AP includes acute edematous pancreatitis (AEP) and acute necrotizing pancreatitis (ANP). AEP has moderate clinical symptoms, and often doesn’t require treatment; whereas ANP has severe clinical symptoms. ANP easily causes complications and sequelae, and may be life-threatening (6).

Obesity is considered an independent risk factor for the development of severe AP (7,8); however, the underlying mechanism remains unknown. Resistin is a newly identified peptide hormone, secreted specifically by adipocytes (9). Resistin can cause obesity and hypertriglyceridemia, due to its association with insulin resistance (10). Previous studies have revealed that resistin is also an important cytokine in inflammatory reactions, and the regulation of other cytokines (11,12). Resistin levels are increased in the pancreatic tissues of patients with AP, and the increased expression has been shown to correlate with the severity of AP (13–15). Thus, resistin may play an important role in the pathogenesis of AP. The aim of the present study was to investigate the association between resistin expression and AP, in order to provide a novel target for the treatment of pancreatitis.

## Materials and methods

### Animal grouping and modeling

A total of 40 healthy male Sprague-Dawley rats (eight weeks old, weighing 200–250 g), provided by the Experimental Animal Center of Nanjing Medical University (Nanjing, China), were housed in a specific-pathogen-free room at a constant temperature of 22–28°C and a relative humidity of 40–70%. The rats were divided at random into four groups (n=10 for each group), which included the control, sham-operated, AEP and ANP groups. The rats were fasted for 12 h, but had free access to water. Caerulein (40 μg/kg; Sigma-Aldrich, Steinheim, Germany) was administered to the rats in the AEP group twice by intraperitoneal injection, with an interval of 1 h. At 12 h after the first injection, a successful model of AEP was established. Rats in the ANP group were treated with arginine using the same method (2 g/kg; Sigma-Aldrich). The ANP rat model was established successfully at 24 h after the first injection. Rats in the sham-operated group were treated identically, but with an equivalent dose of physiological saline. Rats in the control group were fasted and received no further treatment. Following the successful establishment of the models, all the rats were anesthetized with 3% pentobarbital sodium (30 mg/kg) by intraperitoneal injection, and weighed. This study was conducted in strict accordance with the recommendations in the Guide for the Care and Use of Laboratory Animals of the National Institutes of Health. The animal use protocol was reviewed and approved by the Institutional Animal Care and Use Committee of Nanjing Medical University.

### Blood and specimen collection

The abdominal cavity of each rat was opened for blood sample collection from the inferior vena cava and removal of the pancreas. Serum was isolated from the blood samples by centrifugation at 1,409 × g for 10 min at 4°C, and was stored at −80°C. The pancreases were weighed and fixed in formalin for histopathological examination.

### Measurement of serum amylase

According to a reported method (16), serum amylase levels were measured using an Hitachi-7150 automatic biochemistry analyzer (Hitachi Corp., Tokyo, Japan) according to the manufacturer’s instructions.

### Pancreatic histopathology scoring

The pancreases were fixed with 10% neutral buffered formalin for 18–24 h, they were then dehydrated and embedded in paraffin. The samples were cut into 4-μm sections, deparaffinized and stained with hematoxylin-eosin. The sections were observed under a light microscope (Olympus, Tokyo, Japan) and scored histopathologically, according to the Schmid scoring criteria (17).

### ELISA

ELISA kits specific for serum resistin, C-reactive protein (CRP), tumor necrosis factor-α (TNF-α) and interleukin (IL)-1β were purchased from R&D Systems, Inc. (Minneapolis, MN, USA). The tests were performed following the manufacturer’s instructions.

### Immunohistochemistry

The paraffin-embedded sections were roasted in an oven at 59°C overnight, and were then immersed twice in xylene, for 15 min each time. The sections were successively immersed in 100%, 95%, 90% and 85% ethanol for 1 min, and then with phosphate-buffered saline three times, for 5 min each time. The endogenous peroxidase activity was blocked with 1% H_2_O_2_ for 15 min, followed by antigen retrieval in citrate buffer (pH 6.0). Following cooling to room temperature, the sections were incubated overnight with a polyclonal rabbit anti-rat resistin antibody (1:200 dilution; cat. no. A00100-01; Beijing Aidibo Biological Technology Co. Ltd., Beijing, China) at 4°C, and then treated with a horseradish peroxidase-conjugated mouse anti-rabbit immunoglobulin G secondary antibody (1:200 dilution; cat. no. A0303; Shanghai Long Island Antibody Diagnostic Reagents Co., Ltd., Shanghai, China) for 30 min at room temperature. Reactivity was detected with a diaminobenzidine kit (Shanghai Long Island Antibody Diagnostic Reagents Co., Ltd.), and the nuclei were counterstained with hematoxylin.

### RNA extraction and quantitative polymerase chain reaction (PCR)

Pancreatic tissues were dissolved in TRIzol reagent (Invitrogen Life Technologies, Carlsbad, CA, USA) and the total RNA was extracted and reverse transcribed using a PrimeScript RT reagents kit (Invitrogen Life Technologies), according to the manufacturer’s instructions. The cDNA concentration and purity were subsequently measured using an ND-1000 ultraviolet spectrophotometer (NanoDrop Technologies, Inc., Wilmington, DE, USA).

Quantitative PCR was performed with an ABI 7500 Real-time PCR System (Applied Biosystems Life Technologies, Carlsbad, CA, USA), using the SYBR Green PCR Master kit (Sigma-Aldrich). The PCR conditions were set as follows: 95°C for 1 min; 95°C for 15 sec, 60°C for 15 sec, and 72°C for 45 sec (40 cycles); followed by 95°C for 15 sec, 60°C for 60 sec and 99°C for 15 sec. The following gene-specific primer pairs were used: Resistin forward, 5′-TGCCAGTGCGGAAGC ATAGA-3′ and reverse, 5′-TCCAGACCCTACTCTCGTTT-3′; β-actin forward, 5′-ATACTCCTGCTTGCTGATCC-3′ and reverse, 5′-CCTGTACGCCAACACAGTGC-3′. The PCR product sizes for resistin and β-actin were 196 and 201 bp, respectively.

### Statistical analysis

Statistical analysis was performed with the SPSS v13.0 software program (SPSS, Inc., Chicago, IL, USA). Measurement data are presented as the mean ± standard deviation and were compared using the t test or Mann-Whitney U test. Numeration data were analyzed using the χ^2^ test. Pearson’s correlation analysis was used to analyze the associations among the levels of serum resistin, CRP, TNF-α and IL-1β and the pancreatic histopathological scores. For all the tests, two-sided P<0.05 was considered to indicate a statistically significant difference.

## Results

### Serum amylase, pancreas/body weight ratio and histopathological changes

Serum amylase levels in the rats from the AEP and ANP groups were significantly higher compared with those in the normal control and sham-operated groups (P<0.01). Furthermore, serum amylase levels in the rats from the ANP group were significantly higher compared with the AEP group (P<0.05). Rats in the AEP group exhibited a small amount of effusion in the abdominal cavity and their pancreases were evidently swollen. Comparatively, rats in the ANP group exhibited greater effusion in the abdominal cavity and their pancreases were presented with evident swelling, sporadic ecchymosis and regional saponification spots. Pancreas weight in the AEP and ANP group rats increased markedly, contributing to the increased pancreas/body weight ratios. When compared with the normal control and sham-operated groups, the pancreas/body weight ratios in the AEP and ANP groups increased significantly (P<0.01), particularly in the ANP group, which exhibited a statistically significant difference when compared with the AEP group (P<0.05). Histopathologically, pancreases in the AEP group presented with evident edema, enlarged interlobular septum, inflammatory cell infiltration and acinus edema. In addition to these changes, pancreases in the ANP group also presented with regional necrosis, lobular structural damage and intraparenchymal hemorrhage. Pancreases in the normal control and sham-operated groups exhibited no significant histopathological changes. Through scoring the histopathological changes according to the Schmid scoring criteria, the ANP group score was shown to be notably higher compared with the AEP group (P<0.05), while both were markedly higher compared with the normal control and sham-operated groups (P<0.01; Table I).

### Levels of serum resistin, CRP, TNF-α and IL-1β

Serum resistin, CRP, TNF-α and IL-1β levels were all elevated in the AEP and ANP groups, as compared with those in the normal control and sham-operated groups (P<0.01). In addition, the levels were significantly higher in the ANP group compared with the AEP group (P<0.05; Table II).

### Correlation analysis

Pearson’s correlation analysis was performed to analyze the correlations among the levels of serum resistin, CRP, TNF-α and IL-1β and the pancreatic histopathological scores. The serum resistin level was found to positively correlate with the serum CRP (r=0.711, P<0.01), TNF-α (r=0.871, P<0.01) and IL-1β levels (r=0.794, P<0.01). Furthermore, the serum resistin level was shown to positively correlate with the pancreatic histopathological scores (r=0.812, P<0.01), as were the serum levels of CRP, TNF-α and IL-1β (r=0.796, 0.899 and 0.788, respectively, P<0.01).

### Location of resistin

**Figure 1:**
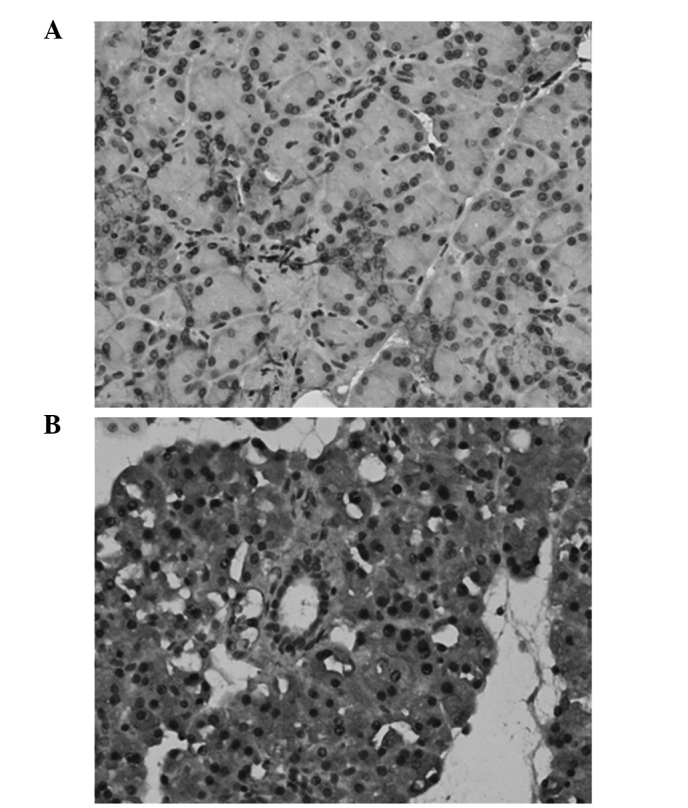
Expression of resistin in the pancreatic tissues of the (A) AEP and (B) ANP group rats (hematoxylin-eosin; magnification, ×400). AEP, acute edematous pancreatitis; ANP, acute necrotizing pancreatitis.

The location of resistin in the pancreatic tissues was detected by immunohistochemical analysis. Resistin was shown to be primarily located in the cytoplasm of the pancreatic acinar cells and strongly expressed in the islet cells. The expression of resistin was increased markedly in the pancreatic tissues of the rats in the AEP and ANP groups (Fig. 1).

### Resistin mRNA expression

The mRNA expression levels of resistin in the pancreatic tissues of the rats in the ANP and AEP groups were significantly higher compared with the control and sham-operated groups (P<0.01). In addition, mRNA expression was significantly higher in the ANP group compared with the AEP group (P<0.05; Table III).

## Discussion

AP is a common clinical disease. Although the etiology and pathogenesis are not yet fully understood, the pathogenesis of AP is associated with a disordered blood circulation and cell apoptosis in the pancreas (18,19).

The present study revealed that serum resistin levels, as well as resistin mRNA and protein expression, in the pancreatic tissues increased significantly in the AEP and ANP groups, as compared with those in the normal control rats (P<0.01). These results indicated that resistin in the pancreatic tissues had been activated and was associated with the degree of inflammation. Moreover, the mRNA and protein expression levels of resistin increased more notably in the ANP group compared with the AEP group, indicating that resistin is activated in the pathogenesis of AP and is involved in pancreatic tissue damage.

In the pathogenesis of AP, particularly in ANP, patients may develop multiple organ dysfunction syndrome, the mechanism of which is hypothesized to be associated with systemic inflammatory response syndrome (SIRS). SIRS is considered to be an inflammatory cascade effect involving cytokines, immune cells and the complement system. Various pathogenic factors damage the pancreas glandular cells and activate immature trypsin, which may further damage the autologous pancreatic tissues in an autocrine manner and activate monocyte-macrophage cells. Cytokines and chemokines released from monocyte-macrophage cells recruit inflammatory cells into the pancreas and other organs, including the lungs, kidneys and liver. Consequently, the inflammatory cells release more cytokines, oxygen free radicals and nitric oxide synthase, which may cause a cascade effect and lead to a vicious circle (20). TNF-α and IL-1β are two of the first cytokines to increase in the pathogenesis of AP, which can initiate a variety of cytokines to promote the formation of pancreatic tissue edema, hemorrhage, necrosis and systemic toxemia. In addition, these cytokines may act on vascular endothelial cells to induce systemic symptoms, such as shock, and increase the deterioration of pancreatitis (21). Moreover, IL-1β is an important factor in sustained pancreatic necrosis and systemic deterioration. IL-1β induces the release of TNF-α and initiates a feedback loop that plays an important role in the onset and development of inflammation (22). Thus, TNF-α and IL-1β are hypothesized to be associated with the progression of AP, and inhibiting their activity may reduce the severity of AP and improve the survival rate in rats (23,24).

Norman *et al* (22) confirmed that the mRNA and protein expression levels of TNF-α and IL-1β in the pancreatic tissues increased significantly in the early stages of AP; however, the increase was delayed in other remote organs. In the damaged organs, TNF-α and IL-1β levels increased significantly and were associated with the degree of organ damage (25).

The present study also demonstrated that serum TNF-α and IL-1β levels were significantly elevated in the pathogenesis of AEP and ANP, particularly in ANP. In addition, the levels were found to correlate with the severity of pancreatitis. Furthermore, serum resistin levels were shown to positively correlate with the serum TNF-α and IL-1β levels, indicating that resistin activation stimulated the release of TNF-α and IL-1β. The underlying mechanism may involve the activation of nuclear factor-κB by resistin, which subsequently stimulates the release of TNF-α and IL-1β, thereby increasing the inflammation of the pancreas. Thus, overproduction of obesity-related circulating resistin and associated low-grade inflammation may result in mild injury to pancreatic acini, increasing the risk and severity of AP (26,27).

CRP is an acute phase protein that is synthesized in the liver and can react to the pneumococcal polysaccharide C. The functions of CRP include binding nucleic acids and phosphatidyl choline, activating the complement system and promoting phagocytosis and immunoregulation. In addition, CRP is a very sensitive indicator for acute inflammation, particularly in AP. Thus, CRP has been used to analyze the severity of AP and evaluate the prognosis of patients with AP (28).

In the present study, the levels of CRP were shown to positively correlate with the pathological scores of AP. In addition, the serum resistin levels were found to correlate with the CRP levels and with the histopathological scores, indicating that resistin may also be used as an indicator for evaluating the severity of AP and the prognosis of patients with AP. Inflammatory cytokines, including IL-6, TNF-α and leptin, are known to be important inducers of CRP (29). With regard to the increased expression of resistin observed in AP, and the associations with CRP and the severity of AP, it was hypothesized that resistin may also be a CRP inducer.

In conclusion, resistin was demonstrated to be activated in the pathogenesis of AP and involved in the injury process of pancreatic tissues. The expression of resistin was found to be associated with the severity of the damage in the pancreatic tissues. Therefore, resistin may be used as an indicator to assess the severity and prognosis of AP, and the hormone may prove a prime target for future therapeutic interventions.

## Figures and Tables

**Table I:** Serum amylase, ratio of pancreas/body weight and histopathological scores in the four groups.

Groups	Cases (n)	Amylase (U/l)	Pancreas/body weight ratio (g/kg)	Histopathological scores
Normal control	10	1230.3±58.58	4.24±0.37	0.54±0.05
Sham-operated	10	1442.2±182.76	4.34±0.42	1.1±0.21
AEP	10	4376.70±342.95^a^	8.67±1.43^a^	5.39±0.26^a^
ANP	10	6750.2±321.81^b^	9.33±1.76^b^	7.81±0.28^b^

Data are expressed as the mean ± standard deviation.

a P<0.01, vs. normal control and sham-operated groups;

b P<0.05, vs. AEP group.

AEP, acute edematous pancreatitis; ANP, acute necrotizing pancreatitis.

**Table II:** Serum resistin, TNF-α, IL-1β and CRP levels in the four groups.

Groups	Cases (n)	Resistin (ng/ml)	IL-1β (pg/ml)	TNF-α (pg/ml)	CRP (ng/ml)
Normal control	10	4.79±0.7	106.03±29.38	103.41±18.95	2664.19±150.20
Sham-operated	10	5.13±0.74	108.74±31.03	106.44±21.31	2894.56±165.34
AEP	10	10.21±1.34^a^	184.18±45.24^a^	194.24±44.81^a^	3585.85±63.03^a^
ANP	10	15.14±0.84^b^	349.31±94.54^b^	315.59±37.04^b^	4345.04±244.14^b^

Data are expressed as the mean ± standard deviation.

a P<0.01, vs. normal control and sham-operated groups;

b P<0.05, vs. AEP group.

IL-1β, interleukin-1β; TNF-α, tumor necrosis factor-α; CRP, C-reactive protein; AEP, acute edematous pancreatitis; ANP, acute necrotizing pancreatitis.

**Table III:** Resistin mRNA expression in the pancreatic tissues of the four groups.

Groups	Cases (n)	mRNA expression of resistin^a^
Normal control	10	0.83±0.05
Sham-operated	10	0.88±0.08
AEP	10	2.04±0.19^b^
ANP	10	3.29±0.30^c^

Data are expressed as the mean ± standard deviation.

a Relative quantitation value.

b P<0.01, vs. normal control group and sham-operated group;

c P<0.05, vs. AEP group.

AEP, acute edematous pancreatitis; ANP, acute necrotizing pancreatitis.

## Abbreviations

AEP: acute edematous pancreatitis
ANP: acute necrotizing pancreatitis
AP: acute pancreatitis
SIRS: systemic inflammatory response syndrome
CRP: C-reactive protein
IL: interleukin
TNF-α: tumor necrosis factor-α
PCR: polymerase chain reaction

## References

[1] Castro FS, Nascimento AM, Coutinho IA, Alcazar FR, Mugayar Filho J (2012). Plasmapheresis as a therapeutic approach for hypertriglyceridemia-induced acute pancreatitis. Rev Bras Ter Intensiva.

[2] Thandassery RB, Yadav TD, Dutta U, Appasani S, Singh K, Kochhar R (2013). Dynamic nature of organ failure in severe acute pancreatitis: the impact of persistent and deteriorating organ failure. HPB (Oxford).

[3] Geoffroy PA, Etain B, Henry C, Bellivier F (2012). Combination therapy for manic phases: a critical review of a common practice. CNS Neurosci Ther.

[4] Woo SM, Noh MH, Kim BG (2011). Comparison of serum procalcitonin with Ranson, APACHE-II, Glasgow and Balthazar CT severity index scores in predicting severity of acute pancreatitis. Korean J Gastroenterol.

[5] Takeda K, Yokoe M, Takada T (2010). Assessment of severity of acute pancreatitis according to new prognostic factors and CT grading. J Hepatobiliary Pancreat Sci.

[6] Wu BU, Banks PA (2013). Clinical management of patients with acute pancreatitis. Gastroenterology.

[7] Sempere L, Martinez J, de Madaria E (2008). Obesity and fat distribution imply a greater systemic inflammatory response and a worse prognosis in acute pancreatitis. Pancreatology.

[8] Papachristou GI, Clermont G, Sharma A, Yadav D, Whitcomb DC (2007). Risk and markers of severe acute pancreatitis. Gastroenterol Clin North Am.

[9] Coelho M, Oliveira T, Fernandes R (2013). Biochemistry of adipose tissue: an endocrine organ. Arch Med Sci.

[10] Steppan CM, Bailey ST, Bhat S (2001). The hormone resistin links obesity to diabetes. Nature.

[11] Hsu CL, Lin YJ, Ho CT, Yen GC (2013). The inhibitory effect of pterostilbene on inflammatory responses during the interaction of 3T3-L1 adipocytes and RAW 264.7 macrophages. J Agric Food Chem.

[12] Minn AH, Patterson NB, Pack S (2003). Resistin is expressed in pancreatic islets. Biochem Biophys Res Commun.

[13] Daniel P, Leśniowski B, Mokrowiecka A, Jasińska A, Pietruczuk M, Małecka-Panas E (2010). Circulating levels of visfatin, resistin and pro-inflammatory cytokine interleukin-8 in acute pancreatitis. Pancreatology.

[14] Leśniowski B, Kumor A, Jasińska A, Daniel P, Pietruczuk M, Małecka-Panas E (2007). Resistin - a new laboratory marker useful in diagnosis of acute pancreatitis?. Pol Merkur Lekarski.

[15] Schäffler A, Landfried K, Völk M, Fürst A, Büchler C, Schölmerich J, Herfarth H (2007). Potential of adipocytokines in predicting peripancreatic necrosis and severity in acute pancreatitis: pilot study. J Gastroenterol Hepatol.

[16] Stefanutti C, Labbadia G, Morozzi C (2013). Severe hypertriglyceridemia-related acute pancreatitis. Ther Apher Dial.

[17] Schmidt J, Rattner DW, Lewandrowski K, Compton CC, Mandavilli U, Knoefel WT, Warshaw AL (1992). A better model of acute pancreatitis for evaluating therapy. Ann Surg.

[18] Cruz-Santamaría DM, Taxonera C, Giner M (2012). Update on pathogenesis and clinical management of acute pancreatitis. World J Gastrointest Pathophysiol.

[19] Charbonney E, Nathens AB (2008). Severe acute pancreatitis: a review. Surg Infect (Larchmt).

[20] Mitchell RMS, Byrne MF, Baillie J (2003). Pancreatitis. Lancet.

[21] Gómez-Cambronero LG, Sabater L, Pereda J, Cassinello N, Camps B, Viña J, Sastre J (2002). Role of cytokines and oxidative stress in the pathophysiology of acute pancreatitis: therapeutical implications. Curr Drug Targets Inflamm Allergy.

[22] Norman JG, Fink GW, Messina J, Carter G, Franz MG (1996). Timing of tumor necrosis factor antagonism is critical in determining outcome in murine lethal acute pancreatitis. Surgery.

[23] Hughes CB, Grewal HP, Gaber LW, Kotb M, El-din AB, Mann L, Gaber AO (1996). Anti-TNFalpha therapy improves survival and ameliorates the pathophysiologic sequelae in acute pancreatitis in the rat. Am J Surg.

[24] Tanaka N, Murata A, Uda K (1995). Interleukin-1 receptor antagonist modifies the changes in vital organs induced by acute necrotizing pancreatitis in a rat experimental model. Crit Care Med.

[25] Norman JG, Fink GW, Denham W (1997). Tissue-specific cytokine production during experimental acute pancreatitis. A probable mechanism for distant organ dysfunction. Dig Dis Sci.

[26] Calabrò P, Cirillo P, Limongelli G (2011). Tissue factor is induced by resistin in human coronary artery endothelial cells by the NF-κB-dependent pathway. J Vasc Res.

[27] Jiang CY, Wang W, Tang JX, Yuan ZR (2013). The adipocytokine resistin stimulates production of proinflammatory cytokines TNF-α and IL-6 in pancreatic acinar cells via NF-κB activation. J Endocrinol Invest.

[28] Mayer JM, Raraty M, Slavin J (2002). Serum amyloid A is a better early predictor of severity than C-reactive protein in acute pancreatitis. Br J Surg.

[29] Bhatia M, Brady M, Shokuhi S, Christmas S, Neoptolemos JP, Slavin J (2000). Inflammatory mediators in acute pancreatitis. J Pathol.

